# Intravenous Thrombolysis with Recombinant Tissue Plasminogen Activator for Ischemic Stroke Patients over 80 Years Old: The Fukuoka Stroke Registry

**DOI:** 10.1371/journal.pone.0110444

**Published:** 2014-10-16

**Authors:** Ryu Matsuo, Masahiro Kamouchi, Haruhisa Fukuda, Jun Hata, Yoshinobu Wakisaka, Junya Kuroda, Tetsuro Ago, Takanari Kitazono, on behalf of the FSR Investigators

**Affiliations:** 1 Department of Medicine and Clinical Science, Graduate School of Medical Sciences, Kyushu University, Fukuoka, Japan; 2 Department of Health Care Administration and Management, Graduate School of Medical Sciences, Kyushu University, Fukuoka, Japan; 3 Center for Cohort Studies, Graduate School of Medical Sciences, Kyushu University, Fukuoka, Japan; University of Münster, Germany

## Abstract

**Objectives:**

The benefit of intravenous recombinant tissue plasminogen activator (rt-PA) therapy for very old patients with acute ischemic stroke remains unclear. The aim of this study was to elucidate the efficacy and safety of intravenous rt-PA therapy for patients over 80 years old.

**Methods:**

Of 13,521 stroke patients registered in the Fukuoka Stroke Registry in Japan from June 1999 to February 2013, 953 ischemic stroke patients who were over 80 years old, hospitalized within 3 h of onset, and not treated with endovascular therapy were included in this study. Among them, 153 patients were treated with intravenous rt-PA (0.6 mg/kg). For propensity score (PS)-matched case-control analysis, 148 patients treated with rt-PA and 148 PS-matched patients without rt-PA therapy were selected by 1∶1 matching with propensity for using rt-PA. Clinical outcomes were neurological improvement, good functional outcome at discharge, in-hospital mortality, and hemorrhagic complications (any intracranial hemorrhage [ICH], symptomatic ICH, and gastrointestinal bleeding).

**Results:**

In the full cohort of 953 patients, rt-PA use was associated positively with neurological improvement and good functional outcome, and negatively with in-hospital mortality after adjustment for multiple confounding factors. In PS-matched case-control analysis, patients treated with rt-PA were still at lower risk for unfavorable clinical outcomes than non-treated patients (neurological improvement, odds ratio 2.67, 95% confidence interval 1.61–4.40; good functional outcome, odds ratio 2.23, 95% confidence interval 1.16–4.29; in-hospital mortality, odds ratio 0.30, 95% confidence interval 0.13–0.65). There was no significant association between rt-PA use and risk of hemorrhagic complications in the full and PS-matched cohorts.

**Conclusions:**

Intravenous rt-PA therapy was associated with improved clinical outcomes without significant increase in risk of hemorrhagic complications in very old patients (aged>80 years) with acute ischemic stroke.

## Introduction

Thrombolysis with intravenous recombinant tissue plasminogen activator (rt-PA) is currently the most effective therapy to improve clinical outcomes in patients with acute ischemic stroke [1]. However, the efficacy and safety of the therapy for very old patients are still controversial.

A large number of studies have reported that functional outcome and in-hospital mortality are unfavorable after thrombolytic therapy in very old patients compared with those in younger patients [2]–[13]. Regarding the safety, it remains unclear whether thrombolysis is associated with an increased risk of adverse events, such as intracranial hemorrhage (ICH), in older patients. Although most previous studies have shown no significant difference in the occurrence of ICH after rt-PA therapy between very old and young patients [2]–[10], [12]–[18], a recent study using the National Impatient Sample Database revealed a significantly higher risk of ICH after thrombolysis in very old versus younger patients [11].

Because of the poor prognosis and hemorrhagic complications after thrombolysis in older adults, randomized controlled trials have typically excluded patients>80 years old [1]. Therefore, evidence for the benefit of thrombolytic therapy in very old individuals remains scarce and the therapy is currently withheld in very old patients with acute ischemic stroke. Recently, the Third International Stroke Trial (IST-3) enrolled ischemic stroke patients up to 6 h from onset without upper age limit to determine whether intravenous thrombolysis with rt-PA is beneficial to a wider range of patients [19]. Consequently, the effect of thrombolysis within 6 h of onset in elderly patients seemed to be at least as large as that in younger patients [20]. As life expectancy is increasing worldwide, data regarding whether to treat very old stroke patients with thrombolysis are important.

Since Asians are more likely to suffer intracranial hemorrhage than non-Asians [21], the effect of rt-PA in very old patients should be validated in different cohorts including an Asian population. In this study, we investigated the association between rt-PA use and clinical outcomes among Japanese patients over 80 years old who were hospitalized within 3 h of onset using a database of acute stroke in the overall cohort and a propensity score (PS)-matched cohort. The aim of this study was to elucidate whether intravenous thrombolysis with rt-PA is efficacious and safe even in very old patients in an Asian setting.

## Methods

### The Fukuoka Stroke Registry

The Fukuoka Stroke Registry (FSR) is a multicenter hospital-based registry in which acute stroke patients within 7 days of onset were enrolled (UMIN Clinical Trial Registry 000000800) [22], [23]. Kyushu University Hospital, National Hospital Organization Kyushu Medical Center, National Hospital Organization Fukuoka-Higashi Medical Center, Fukuoka Red Cross Hospital, St. Mary's Hospital, Steel Memorial Yawata Hospital, and the Japan Labor Health and Welfare Organization Kyushu Rosai Hospital in Fukuoka, Japan participate in this registry (Text S1). Standardized instruments were used to collect demographic characteristics, co-morbidities, laboratory data, and medical histories of the patients.

### Study subjects

Stroke was defined as sudden onset of focal neurological deficits persisting for more than 24 h and classified into ischemic stroke, brain hemorrhage, subarachnoid hemorrhage, or other types of stroke by means of brain imaging (computed tomography [CT] and/or magnetic resonance imaging). In a total of 13,521 patients registered in the FSR retrospective (7387 patients from June 1999 to May 2007) and prospective (6134 patients from June 2007 to February 2013) databases, 11,432 patients were diagnosed as having ischemic stroke. Among them, 972 patients who were aged older than 80 years and hospitalized within 3 h after onset were selected. After excluding 19 patients who underwent endovascular therapy, we included 953 patients (506 patients in retrospective and 447 patients in prospective cohorts) as the full cohort of this study.

**Figure 1:**
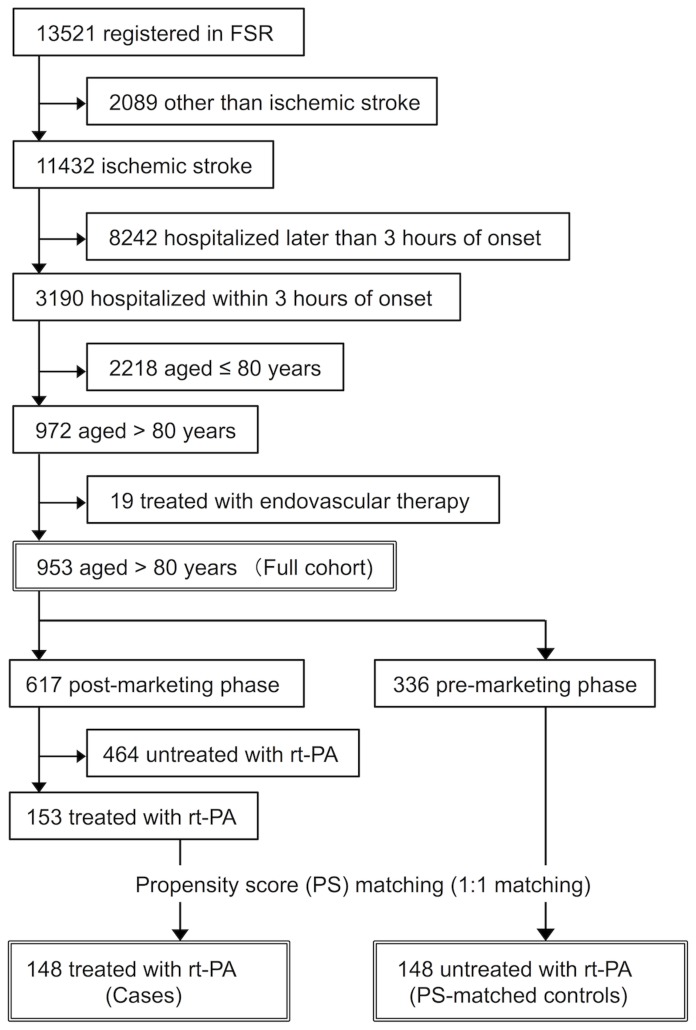
Flow chart of patient selection.

Patients in the full cohort were further divided into two groups according to whether they were admitted before or after approval of intravenous administration of rt-PA (alteplase in Japan). Accordingly, 336 patients were included in the pre-marketing phase (June 1999 to September 2005) and 617 patients in the post-marketing phase (October 2005 to February 2013). In the post-marketing phase, 153 cases were treated with intravenous administration of rt-PA. Patient eligibility for alteplase was determined in accordance with a Japanese guideline [24], and alteplase was administered in 25.9% of patients of all ages who were hospitalized within 3 h from onset in the post-marketing phase. Each patient received alteplase (0.6 mg/kg) intravenously with 10% given as a bolus within 3 h of stroke onset and the remainder was delivered through continuous intravenous infusion over 1 h. PS-matched controls were selected from ischemic stroke patients who were aged older than 80 years and hospitalized within 3 h of onset during the pre-marketing phase. After calculating PS, 148 cases and 148 PS-matched controls were selected for PS-matched analysis (Figure 1).

### Clinical assessment

Hypertension was defined as systolic blood pressure ≥140 mm Hg or diastolic blood pressure ≥90 mm Hg in the chronic stage, or as a previous history of treatment with antihypertensive drugs. Dyslipidemia was defined as a low-density lipoprotein-cholesterol level ≥3.62 mmol/L, high-density lipoprotein-cholesterol level ≤1.03 mmol/L, triglyceride level ≥1.69 mmol/L, or a previous history of treatment with a lipid-lowering drug. Diabetes mellitus was determined by either the diagnostic criteria of the Japan Diabetes Society in the chronic stage or based on a medical history of diabetes. Atrial fibrillation was diagnosed based on electrocardiographic findings on admission or during hospitalization, or a previous history of atrial fibrillation. Smoking was defined as current or former cigarette smoking, and alcohol intake was defined as habitual consumption of alcohol beverages before onset of stroke. Chronic kidney disease was defined as an estimated glomerular filtration rate (eGFR) <60 mL/min per 1.73 m^2^, in which eGFR was determined using the equation proposed by the Japanese Society of Nephrology as follows: eGFR (mL/min per 1.73 m^2^) = 194×(serum creatinine [mg/dL])^1.094^×age [year])^−0.287^×0.739 (if female) [25]. Ischemic heart disease was defined as a previous history of angina pectoris, myocardial infarction, and percutaneous coronary intervention or coronary artery bypass graft surgery. Pre-stroke independency was defined as a modified Rankin Scale (mRS) score of 0–1 before stroke onset.

### Study outcomes

Neurological severity was scaled by the National Institutes of Health Stroke Scale (NIHSS) score on admission and at discharge. The average length of stay was 32.9±22.3 days. Neurological improvement was defined as a ≥4 point decrease in the NIHSS score during hospitalization or a NIHSS score of 0 at discharge [26]. In-hospital mortality was defined as all causes of death during hospitalization. To evaluate the short-term functional outcome, post-stroke functional impairment at discharge was graded using an mRS. A good outcome was defined as functional independence (mRS score of 0–2). To evaluate the safety of rt-PA, three types of hemorrhagic complications were assessed during hospitalization: any ICH, symptomatic ICH, and gastrointestinal bleeding. ICH was determined by using CT irrespective of neurological worsening, and symptomatic ICH was defined as any type of CT-documented hemorrhage either within the infarct area or in other areas, concomitant with any neurological worsening. Gastrointestinal bleeding was defined as any episodes of hematemesis or melena during hospitalization.

### PS matching and statistical analysis

Baseline characteristics were compared by the χ^2^ test or the McNemar-Bowker test, the unpaired or paired *t* test, and the Wilcoxon rank sum test or the Wilcoxon signed rank test, as appropriate. In the full cohort model, logistic regression analyses were used to estimate multivariable-adjusted odds ratios and 95% confidence intervals for study outcomes. Statistical analyses were performed using JMP software ver. 11 (SAS Institute, Cary, NC, USA). The probability of receiving rt-PA therapy (PS) was calculated for the rt-PA-treated and the non-treated patients using a logistic regression model. Eighteen covariates, including age, sex, hypertension, dyslipidemia, diabetes, atrial fibrillation, smoking, drinking, ischemic heart disease, chronic kidney disease, pre-stroke independency, previous stroke, antithrombotic therapy before onset, cardioembolic stroke, NIHSS on admission, admission within 2 h from onset, systolic blood pressure, and length of hospital stay were used to generate PS. After PS generation, patients treated with rt-PA and those untreated with rt-PA underwent 1∶1 nearest neighbor (Greedy-type) matching of the standard deviation of the logit of the PS with a caliper width of 0.25. Matching was performed without replacement, and unpaired cases and controls not meeting matching criteria were excluded. Each PS-derived matched pair was assigned a unique pair ID. A total of 148 matched pair IDs were selected. Calculation of PS and 1∶1 matching were performed using STATA13 (StataCorp LP, College Station, TX, USA). In the PS-matched cohort model, odds ratios of clinical outcomes were calculated after matching using conditional logistic regression analysis. Probability values of <0.05 were considered statistically significant.

### Ethics Statement

The study design was approved by the institutional review boards and ethics committees of all hospitals (Kyushu University Hospital Institutional Review Board, National Hospital Organization Kyushu Medical Center Institutional Review Board, National Hospital Organization Fukuoka-Higashi Medical Center Institutional Review Board, Fukuoka Red Cross Hospital Institutional Review Board, St. Mary's Hospital Institutional Review Board, Steel Memorial Yawata Hospital Institutional Review Board, and the Japan Labor Health and Welfare Organization Kyushu Rosai Hospital Institutional Review Board). The study was conducted according to the principles expressed in the Declaration of Helsinki. For the retrospective database, we reviewed medical records of all consecutive patients with acute stroke who were hospitalized in participating hospitals within 24 h of onset. The institutional review boards waived the requirement for obtaining informed consent from patients in retrospective database, because medical information had been collected as part of routine clinical care and processed into anonymized database. In prospective database, written informed consent was obtained from each patient or his/her proxy [22], [23].

## Results

### Background characteristics in the full cohort

Background characteristics of ischemic stroke patients who were over 80 years old and hospitalized within 3 h of onset are shown according to treatment with or without intravenous administration of rt-PA (Table 1). The frequency of atrial fibrillation, pre-stroke independency, cardioembolic stroke, and admission within 2 h from onset was significantly higher, but the prevalence of previous stroke was less frequent, in rt-PA-treated versus rt-PA non-treated patients. Systolic blood pressure on admission was lower and neurological symptoms were more severe in rt-PA treated patients compared with those without rt-PA. The numbers of patients aged 81–85 years, 86–90 years, 91–95 years and ≥96 years were 478 (50.2%), 326 (34.2%), 119 (12.5%), and 30 (3.1%), respectively. In the post-marketing period, 307 (49.8%), 206 (33.4%), 78 (12.6%), and 26 (4.2%) patients were aged 81–85 years, 86–90 years, 91–95 years and ≥96 years, respectively, and the frequencies of patients treated with rt-PA therapy were 23.4%, 25.7%, 25.6%, and 30.8% in each age range, respectively.

**Table 1:** Baseline characteristics in rt-PA-treated and non-treated patients in the full cohort.

	rt-PA treated	rt-PA non-treated	P
	n = 153	n = 800	
Age, years, median (IQR)	86 (84–89)	85 (83–88)	0.09
Female, n (%)	96 (62.7)	489 (61.1)	0.71
Risk factors, n (%)			
Hypertension	117 (76.5)	602 (75.3)	0.75
Dyslipidemia	47 (30.7)	200 (25.0)	0.15
Diabetes	28 (18.3)	187 (23.4)	0.16
Atrial fibrillation	94 (61.4)	396 (49.5)	0.007
Smoking	41 (26.8)	198 (24.8)	0.59
Drinking	26 (17.0)	125 (15.6)	0.67
Chronic kidney disease, n (%)	83 (54.2)	446 (55.8)	0.73
Pre-stroke independency, n (%)	108 (70.6)	474 (59.3)	0.007
Previous stroke, n (%)	29 (19.0)	243 (30.4)	0.003
Previous ischemic heart disease, n (%)	38 (24.8)	165 (20.6)	0.25
Pre-stroke antithrombotic therapy, n (%)	76 (49.7)	352 (44.0)	0.20
Cardioembolic stroke, n (%)	108 (70.6)	406 (50.8)	<0.001
Admission within 2 hours from onset, n (%)	136 (88.9)	586 (73.3)	<0.001
Systolic blood pressure, mmHg, mean ± SD	156±27	161±31	0.045
NIHSS on admission, median (IQR)	16 (9.5–21)	9 (3–16.75)	<0.001
Length of hospital stay, days, mean ± SD	31.8±17.6	33.1±25.1	0.53

IQR: interquartile range. Pre-stroke independency was defined as mRS 0–1 before onset.

### Treatment with rt-PA and clinical outcomes in the full cohort

Without adjustment for background characteristics, the severity of neurological symptoms at discharge was comparable between rt-PA treated (NIHSS score: median 7, interquartile range [IQR] 1–16) and non-treated (NIHSS score: median 6, IQR 1–15.75; P = 0.73) patients. Further, mRS at discharge was not different between patients with (median 4, IQR 2–5) and without (median 4, IQR 3–5; P = 0.27) intravenous rt-PA.

Next, we performed multivariable logistic regression analysis to adjust for possible confounding factors. Age- and sex- or multivariable-adjusted odds ratios for each clinical outcome are shown in Table 2. Consequently, use of rt-PA was positively associated with neurological improvement and good functional outcome and inversely with in-hospital mortality after adjustment for confounders. The age- and sex-adjusted risk of any ICH was higher in the rt-PA treated group than the rt-PA non-treated group, but this association failed to reach significance after adjustment for multiple confounding factors. rt-PA treatment was not associated with risk of symptomatic ICH and gastrointestinal bleeding.

**Table 2:** Association between rt-PA and clinical outcomes in the full cohort.

	Events (%)		Age- and sex-adjusted			Multivariable-adjusted		
	rt-PA-treated n = 153	rt-PA non-treated n = 800	OR	95% CI	P	OR	95% CI	P
Neurological improvement	95 (62.1)	295 (36.9)	2.90	2.03–4.18	<0.001	2.60	1.77–3.84	<0.001
Good functional outcome	39 (25.5)	199 (24.9)	1.12	0.74–1.69	0.58	3.09	1.66–5.83	<0.001
In-hospital mortality	13 (8.5)	97 (12.1)	0.66	0.34–1.17	0.16	0.31	0.15–0.61	<0.001
Any ICH	19 (12.4)	49 (6.1)	2.20	1.23–3.81	0.009	1.75	0.96–3.09	0.07
Symptomatic ICH	8 (5.2)	23 (2.9)	1.95	0.80–4.29	0.13	1.82	0.73–4.16	0.19
Gastrointestinal bleeding	4 (2.6)	13 (1.6)	1.72	0.48–4.97	0.37	1.56	0.42–3.46	0.48

OR: odds ratio, CI: confidence interval, ICH: intracranial hemorrhage. Neurological improvement was defined as a ≥4 point decrease in the NIHSS score during hospitalization or a NIHSS score of 0 at discharge. Good functional outcome was defined as an mRS score of 0–2 at discharge. Multivariable logistic model for neurological improvement, good functional outcome, and in-hospital mortality included age, sex, hypertension, dyslipidemia, diabetes, atrial fibrillation, smoking, drinking, chronic kidney disease, pre-stroke independency, previous stroke, previous ischemic heart disease, pre-antithrombotic therapy, cardioembolic stroke, admission within 2 h of onset, and NIHSS on admission. Multivariable logistic model for ICH, symptomatic ICH, and gastrointestinal bleeding included age, sex, systolic blood pressure, diabetes, chronic kidney disease, and NIHSS on admission.

### Background characteristics in the PS-matched cohort

To control confounding factors by indication, we calculated PS of rt-PA-treated cases and randomly selected one control that had a similar PS to each case. The mean standardized difference in covariates decreased from 19.1% (range 1.2–75.7%) before matching to 4.3% (range 0–12.7%) after matching (Figure S1). After 1∶1 matching, covariates were statistically indistinguishable between rt-PA-treated cases and PS-matched rt-PA-non-treated controls (Table 3).

**Table 3:** Baseline characteristics in rt-PA-treated and non-treated patients in the PS-matched cohort.

	rt-PA treated	rt-PA non-treated	P
	n = 148	n = 148	
Age, years, median (IQR)	86 (84–89)	86 (83–90)	0.88
Female, n (%)	93 (62.8)	99 (66.9)	0.47
Risk factors, n (%)			
Hypertension	112 (75.7)	112 (75.7)	1.00
Dyslipidemia	43 (29.1)	42 (28.4)	0.90
Diabetes	28 (18.9)	33 (22.3)	0.47
Atrial fibrillation	91 (61.5)	85 (57.4)	0.48
Smoking	39 (26.4)	38 (25.7)	0.89
Drinking	24 (16.2)	25 (16.9)	0.88
Chronic kidney disease, n (%)	81 (54.7)	84 (56.8)	0.73
Pre-stroke independency, n (%)	103 (69.6)	105 (70.9)	0.80
Previous stroke, n (%)	29 (19.6)	27 (18.2)	0.77
Previous ischemic heart disease, n (%)	34 (23.0)	38 (25.7)	0.59
Pre-stroke antithrombotic therapy, n (%)	72 (48.6)	66 (44.6)	0.48
Cardioembolic stroke, n (%)	105 (70.9)	96 (64.9)	0.26
Admission within 2 hours from onset, n (%)	131 (88.5)	127 (85.8)	0.49
Systolic blood pressure, mmHg, mean ± SD	156±28	157±30	0.87
NIHSS on admission, median (IQR)	16 (9.25–21)	16 (9–21)	0.86
Length of hospital stay, days, mean ± SD	31.8±17.8	31.8±23.0	0.98

IQR: interquartile range. Pre-stroke independency was defined as an mRS score of 0–1 before onset.

### Treatment with rt-PA and clinical outcomes in the PS-matched cohort

In the PS-matched cohort, neurological symptoms were less severe at discharge in rt-PA-treated (NIHSS score: median 7, IQR 1–16) compared with PS-matched non-treated (NIHSS score: median 13, IQR 4–26; P<0.001) patients. Further, mRS at discharge was significantly lower in rt-PA-treated (median 4, IQR 2.25–5) compared with non-treated (median 5, IQR 4–5; P<0.001) patients. Incident rates and odds ratios of clinical outcomes and hemorrhagic complications are shown in Table 4. Intravenous rt-PA therapy was associated positively with neurological improvement and good functional outcome, and negatively with in-hospital mortality. The frequencies of hemorrhagic complications, including any ICH, symptomatic ICH, and gastrointestinal bleeding were not statistically different between rt-PA-treated and PS-matched non-treated patients.

**Table 4:** Association between rt-PA and clinical outcomes in the PS-matched cohort.

	Events (%)		Unadjusted		
	rt-PA-treated, n = 148	rt-PA non-treated, n = 148	OR	95% CI	P
Neurological improvement	91 (61.5)	56 (37.8)	2.67	1.61–4.40	<0.001
Good functional outcome	37 (25.0)	21 (14.2)	2.23	1.16–4.29	0.02
In-hospital mortality	13 (8.8)	32 (21.6)	0.30	0.13–0.65	0.003
Any ICH	17 (11.5)	12 (8.1)	1.45	0.68–3.13	0.34
Symptomatic ICH	7 (4.7)	6 (4.1)	1.17	0.39–3.47	0.78
Gastrointestinal bleeding	4 (2.7)	3 (2.0)	1.33	0.30–5.96	0.71

OR: odds ratio, CI: confidence interval, ICH: intracranial hemorrhage. Neurological improvement was defined as a ≥4 point decrease in the NIHSS score during hospitalization or a NIHSS score of 0 at discharge. Good functional outcome was defined as an mRS score of 0–2 at discharge.

## Discussion

The main findings of the present study were that in patients aged over 80 years, (1) rt-PA was associated positively with neurological improvement and good functional outcome, and negatively with in-hospital mortality, and (2) there was no association between rt-PA use and hemorrhagic complications, including any ICH, symptomatic ICH, and gastrointestinal bleeding. These data support the idea that thrombolytic therapy using intravenous rt-PA is efficacious without increasing risk of harmful hemorrhagic complications in Japanese patients over 80 years old.

A number of studies have investigated the benefit of thrombolytic therapy in very old patients (aged>80 years) compared with younger patients. However, the majority of studies suggest that clinical outcomes are unfavorable after thrombolytic therapy in very old compared with younger patients [2]–[13]. Recently, we have also reported that poor functional outcome and in-hospital mortality were more prevalent after thrombolytic therapy in older patients compared with younger ones [27]. Because the prognosis is generally poor in older versus younger patients, further understanding of the benefit of thrombolytic therapy among elderly patients is required. Therefore, in the present study, we compared the efficacy and safety of intravenous thrombolysis among patients over 80 years old in the overall cohort and a PS-matched cohort to control confounding factors by indication.

There are few studies that have investigated the efficacy of rt-PA therapy among patients aged>80 years. Alshekhlee et al. reported that the odds ratio of in-hospital mortality was increased to 1.38 (95% confidence interval 1.22–1.58) in patients>80 years old treated with thrombolysis compared with non-treated patients [11]. Sung et al. found no difference in the frequency of home discharges and mRS ≤2 between rt-PA-treated and rt-PA-non-treated patients aged ≥80 years with acute ischemic stroke [17]. However, a recent study using the database of Safe Implementation of Treatment in Stroke-International Stroke Thrombolysis Registry (SITS-ISTR, December 2002 to November 2009) and Virtual International Stroke Trials Archive (VISTA, 1998–2007) showed that improved outcome was maintained in very old patients [28]. Additionally, IST-3 enrolled acute ischemic stroke patients up to 6 h of stroke onset without upper age limit from non-Asian countries. Though 53% were older than 80 years of age and 72% were treated between 3 h and 6 h of onset in IST-3, intravenous thrombolytic therapy seemed to have favorable effects on functional outcome in very old patients [20]. Therefore, age alone may not be a barrier to treatment. In this study, approximately half of patients were distributed in their early eighties. However, intravenous thrombolysis with rt-PA was performed equally throughout all age ranges. Thus, in the clinical setting rt-PA therapy is performed even in extremely old patients irrespective of age. We found that intravenous rt-PA therapy was significantly associated with favorable outcomes after adjustment for possible confounding factors in the full cohort of patients aged over 80 years. Moreover, PS-matched analysis revealed that the relative risk of poor functional outcome and in-hospital mortality was reduced by 13% and 59%, respectively, after intravenous thrombolysis. Our results support the idea that clinical outcomes are improved by thrombolysis even in older patients in an Asian setting.

We also investigated the frequency of hemorrhagic complications to evaluate the safety of thrombolysis using intravenous alteplase. Most previous studies report that the risk of hemorrhagic complications after thrombolysis was not higher in very older patients than in younger subjects [2]–[10], [12]–[18]. By contrast, Alshekhlee et al. reported that use of thrombolysis was associated with increased risk of ICH (odds ratio 9.69, 95% confidence interval 6.25–15.02) in older patients after adjustment for multiple confounding factors [11]. In our study, there was no difference in the incidence of hemorrhagic complications such as symptomatic ICH and gastrointestinal bleeding, suggesting that intravenous thrombolysis may not cause significant harm to very old patients.

In October 2005, the use of intravenous alteplase was approved as a treatment for acute ischemic stroke in Japan. As the Asian population has a higher risk for spontaneous brain hemorrhage [21], the dose of alteplase approved for Japanese patients was as low as 0.6 mg/kg based on data from the Japan Alteplase Clinical Trial (J-ACT) [29]. Nonetheless, the frequency of symptomatic ICH in J-ACT (17%) was comparable to that in elderly patients reported in other observational studies from Europe or North America (3–14%). Low-dose rt-PA might reduce the risk of hemorrhagic complications, but not improve clinical outcomes due to thrombus lysis. Our data suggest that low-dose intravenous alteplase (0.6 mg/kg) provides greater benefit than harm in Japanese patients aged>80 years. The optimal dose and benefit of intravenous rt-PA in very old patients should be validated in other cohorts.

The present study has strengths. The benefit of intravenous thrombolysis in very old patients was evaluated using PS matching. To avoid confounding by indication, controls were selected from those enrolled before approval of intravenous alteplase. Patients in the registry were consecutively enrolled based on the standardized measurement. There are also limitations in our study. First, the number of patients over 80 years old was still small and hemorrhagic events rarely occurred. Second, background characteristics could not be completely adjusted for. A progress in stroke care may have affected the results, although possible confounders were included in multivariable adjustment or PS. Third, the generalizability is problematic, because patients were selected from those admitted to one university hospital and six community hospitals in the restricted region of Japan. Additionally, the dose of rt-PA was lower than that in other countries. External validation is required to confirm our findings.

## Supporting Information

Figure S1 **Distribution of standardized differences before and after 1∶1 matching.**
(PDF)

Text S1 **Fukuoka Stroke Registry.**
(PDF)
