# Supplementary material for: Intravenous Thrombolysis with Recombinant Tissue Plasminogen Activator for Ischemic Stroke Patients over 80 Years Old: The Fukuoka Stroke Registry
Source: PLoS One. 2014 Oct 16;9(10):e110444. doi: 10.1371/journal.pone.0110444 (PMC4199731; doi:10.1371/journal.pone.0110444)
Supplement: Figure S1 — Distribution of standardized differences before and after 1∶1 matching. (PDF) [file pone.0110444.s001.pdf]

Figure S1. Distribution of standardized differences before and after 1:1 matching

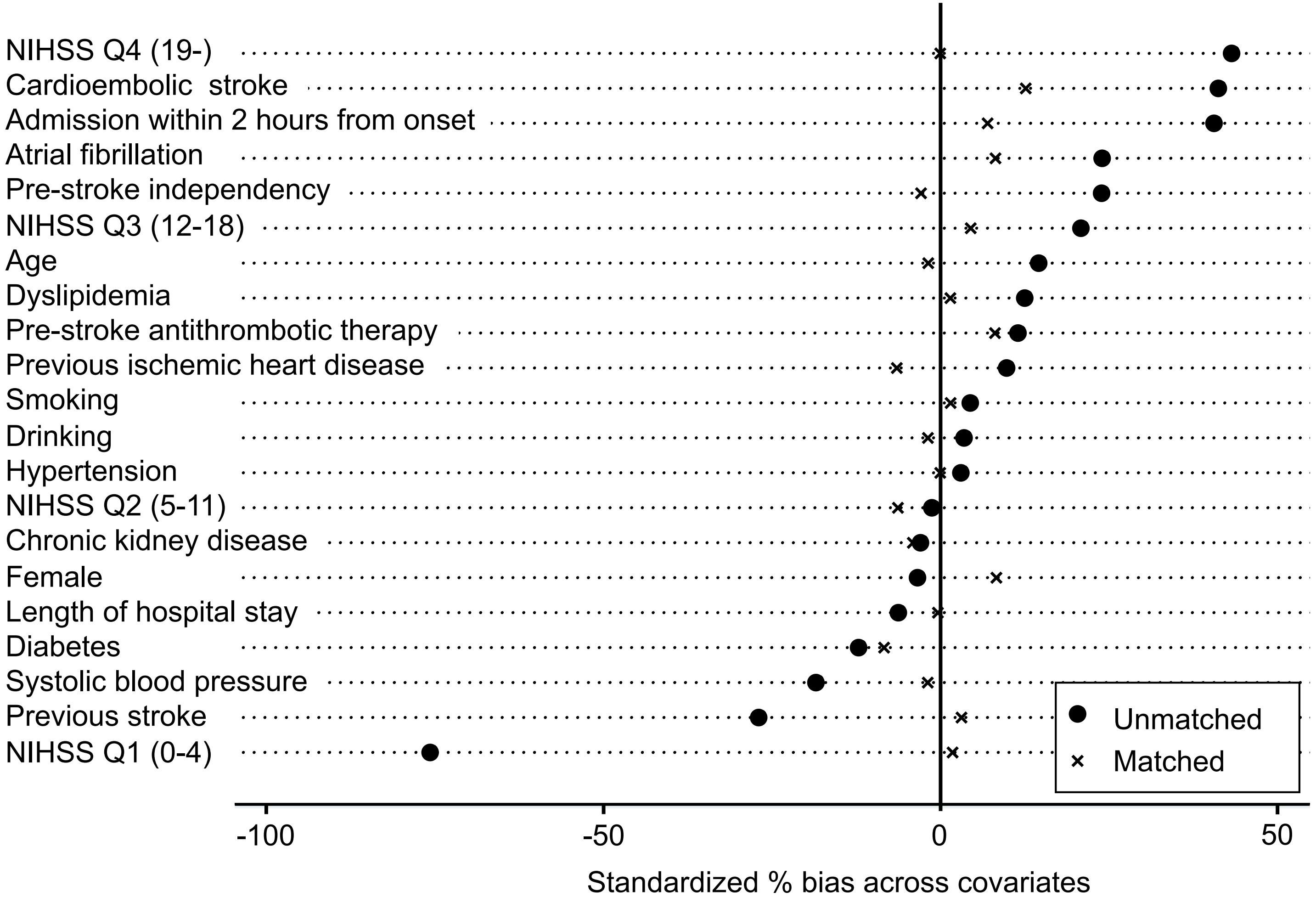

The distribution of standardized differences is shown across covariates before (circles) and after matching (crosses).
