# Supplementary material for: Intravenous Thrombolysis with Recombinant Tissue Plasminogen Activator for Ischemic Stroke Patients over 80 Years Old: The Fukuoka Stroke Registry
Source: PLoS One. 2014 Oct 16;9(10):e110444. doi: 10.1371/journal.pone.0110444 (PMC4199731; doi:10.1371/journal.pone.0110444)
Supplement: Text S1 — Fukuoka Stroke Registry. (PDF) [file pone.0110444.s002.pdf]

Text S1. Fukuoka Stroke Registry

The participating hospitals in the Fukuoka Stroke Registry were the following: Kyushu University Hospital, National Hospital Organization Kyushu Medical Center, National Hospital Organization Fukuoka-Higashi Medical Center, Fukuoka Red Cross Hospital, St. Mary's Hospital, Steel Memorial Yawata Hospital, and the Japan Labor Health and Welfare Organization Kyushu Rosai Hospital. The steering committee included the following: Takao Ishitsuka, MD (Steel Memorial Yawata Hospital), Shigeru Fujimoto, MD (Steel Memorial Yawata Hospital), Setsuro Ibayashi, MD (Seiai Rehabilitation Hospital), Kenji Kusuda, MD (Seiai Rehabilitation Hospital), Shuji Arakawa, MD (Japan Labour Health and Welfare Organization Kyushu Rosai Hospital), Kinya Tamaki, MD (Shin-Yoshizuka Hospital), Seizo Sadoshima, MD (Shin-Yoshizuka Hospital), Katsumi Irie, MD (Hakujyuji Hospital), Kenichiro Fujii, MD (Fukuoka Red Cross Hospital), Yasushi Okada, MD (National Hospital Organization Kyushu Medical Center), Masahiro Yasaka, MD (National Hospital Organization Kyushu Medical Center), Tetsuhiko Nagao, MD (Midorino clinic), Hiroaki Ooboshi, MD (Fukuoka Dental Collage Medical and Dental Hospital), Tsuyoshi Omae, MD (Imazu Red Cross Hospital), Kazunori Toyoda, MD (National Cerebral and Cardiovascular Center), Hiroshi Nakane, MD (National Hospital Organization Fukuoka-Higashi Medical Center), Hiroshi Sugimori, MD (Saga-Ken Medical Centre Koseikan), Kenji Fukuda, MD (St. Mary's Hospital), and Yoshihisa Fukushima, MD (St. Mary's Hospital).
